# Physicochemical properties of dietary phytochemicals can predict their passive absorption in the human small intestine

**DOI:** 10.1038/s41598-017-01888-w

**Published:** 2017-05-16

**Authors:** Sophie N. B. Selby-Pham, Rosalind B. Miller, Kate Howell, Frank Dunshea, Louise E. Bennett

**Affiliations:** 1 Faculty of Veterinary and Agricultural Science, The University of Melbourne, Parkville, 3010 Australia; 2 CSIRO Agriculture and Food, 671 Sneydes Road, Werribee, 3030 Australia; 3 CSIRO Data61, North Ryde, 2113 Australia

## Abstract

A diet high in phytochemical-rich plant foods is associated with reducing the risk of chronic diseases such as cardiovascular and neurodegenerative diseases, obesity, diabetes and cancer. Oxidative stress and inflammation (OSI) is the common component underlying these chronic diseases. Whilst the positive health effects of phytochemicals and their metabolites have been demonstrated to regulate OSI, the timing and absorption for best effect is not well understood. We developed a model to predict the time to achieve maximal plasma concentration (T_max_) of phytochemicals in fruits and vegetables. We used a training dataset containing 67 dietary phytochemicals from 31 clinical studies to develop the model and validated the model using three independent datasets comprising a total of 108 dietary phytochemicals and 98 pharmaceutical compounds. The developed model based on dietary intake forms and the physicochemical properties lipophilicity and molecular mass accurately predicts T_max_ of dietary phytochemicals and pharmaceutical compounds over a broad range of chemical classes. This is the first direct model to predict T_max_ of dietary phytochemicals in the human body. The model informs the clinical dosing frequency for optimising uptake and sustained presence of dietary phytochemicals in circulation, to maximise their bio-efficacy for positively affect human health and managing OSI in chronic diseases.

## Introduction

Chronic diseases are the leading causes of mortality in the world, responsible for 68% of all deaths^1^. Current evidence strongly supports that diets rich in plant foods are associated with reduced risk of chronic diseases such as cardiovascular^2^ and neurodegenerative diseases^3^, obesity^4^, diabetes^5^ and cancer^6^. Oxidative stress and inflammation (OSI) are consistently high in people suffering from chronic diseases^7^. These transient elevated states of OSI can also be associated with daily cycles of activity including meal digestion^8^ and exercise^9^ in healthy individuals. Ingestion of a phytochemical-rich fruit juice or grape extracts can prevent post-prandial OSI induced by a high-fat meal challenge in healthy volunteers^10–12^. Similarly, positive health effects of phytochemicals have been demonstrated to attenuate the OSI associated with exercise in athletes^13, 14^.

Uptake of dietary phytochemicals in the human body and their bioavailability to target cells facilitate their bio-efficacy to protect our health^15^. However, phytochemicals have relatively low bioavailability as they are handled by the body as xenobiotics therefore the presence in the body is transient^16^. Following the ingestion of phytochemicals, some but not all components are absorbed into the circulatory system *via* the small intestine^15^. These phytochemicals may be subjected to metabolism in the liver and their hepatic metabolites are released back into the circulatory system^15^. The phytochemicals that are not absorbed in the small intestine reach the colon whereby substantial structural modification by the colonic microbiota occurs and their microbial metabolites are released back into the circulatory system^16^. The main factors affecting the bioavailability of phytochemicals include chemical structures and dietary intake forms^15^. The chemical heterogeneity of key bioactive phytochemicals within dietary plants results in a broad range of associated time required to reach maximal plasma concentration (T_max_) in the body^17^. For example, green tea flavan-3-ols peak in human plasma within 1–2 hour (h) post ingestion and cleared over the next few hours^18^ whilst maximal levels of tomato lycopene was observed between 15 and 33 h post-ingestion and completely cleared over the next few days^19^. Additionally, dietary intake forms of phytochemicals may also have an impact on their T_max_ in the body^20^. Ellagic acid from a pomegranate extract was reported to have a T_max_ of 0.5–1 h when ingested as liquid form, but 2–3 h when ingested in a solid form^21^. It is possible that previous studies have underestimated the OSI-reducing effects of dietary phytochemicals if blood sampling was performed outside the timespan of T_max_ in the body. For example, no effects of vitamin C supplementation (1 g/d) on plasma biomarkers of OSI were reported after either 1 day or 2 week treatment durations^22^. However, bolus dose of vitamin C given 2 h before exercise prevented exercise-induced OSI^23^. The inconsistency in findings of bio-efficacy of vitamin C could be due to the time of blood sampling that mismatched the short T_max_ of vitamin C (~3 h^24^). The timing of dietary phytochemical consumption relative to OSI challenges (*e.g*., meal or exercise) could be an important factor in understanding and optimising the health benefits of phytochemicals.

Oral bioavailability of phytochemicals can be informed by the application of *in silico* modelling widely used in pharmaceutical sciences^25^ and drug discovery^26^. These models correlate *in vitro* and/or *in vivo* passive absorption of drugs with their chemical structures described by physicochemical properties to predict the absorption of similar compounds^27^. Physicochemical properties of importance in drug absorption include molecular mass (M_r_), lipophilicity (expressed as the logarithm of the partition coefficient between water and 1-octanol, log P), number of hydrogen (H) donors and acceptors^28^, polar surface area (PSA), number of freely-rotatable bonds^29^ and molecular volume^30^. Multiple models have been developed to predict absorption kinetics and bioavailability of pharmaceutical compounds^27^. However, there is currently no such model for predicting T_max_ of dietary phytochemicals from physicochemical properties.

The aim of this study was to determine if T_max_ of dietary phytochemicals in healthy individuals could be predicted from standard physicochemical properties and dietary intake forms. To develop the predictive model, we used a training dataset that modelled the T_max_ of 67 dietary phytochemicals collected from 31 clinical studies of healthy volunteers^18, 19, 21, 24, 31–57^ to their calculated physicochemical properties. To validate the predictive model for dietary phytochemicals, we used an independent phytochemical validation dataset (PCv) containing 108 dietary phytochemicals collected from a further 34 clinical studies^58–91^. We validated the predictive model using pharmaceutical compounds and evaluated the effects of food on the prediction accuracy of the model by using two datasets containing 60 pharmaceutical compounds ingested without food (PHv-fasted)^92–148^ and 38 pharmaceutical compounds ingested with food (PHv-fed)^92–95, 97, 98, 102–104, 106–111, 113, 116, 117, 121, 122, 126, 128, 130–133, 136, 138, 140, 143–146, 148–151^. This study demonstrates that physicochemical properties and dietary intake forms can be used to predict T_max_ of dietary phytochemicals and pharmaceutical compounds when ingested without food.

## Results

### Correlation analysis of the training dataset

The model training dataset contained 11 variables including T_max_, 8 physicochemical properties and 3 categories of dietary intake forms (Supplementary Table S1). The included physicochemical properties were M_r_, log P, PSA, number of freely rotatable bonds, number of H donors, number of H acceptors and molecular volume. As there is a high correlation between variables, multi-collinearity affects the estimation of the coefficients and inflates the standard errors (SE). Therefore, to investigate the relationships between the physicochemical properties in the training dataset, Pearson correlation analyses were performed. Table 1 provides these Pearson’s correlation coefficients (r) with their associated P-values. Significantly high correlations (|r| > 0.75, P < 0.05) were observed between M_r_ and number of freely rotatable bonds (r = 0.772, P < 0.001), M_r_ and molecular volume (r = 0.949, P < 0.001), log P and number of H acceptors (r = −0.755, P < 0.001), number of freely rotatable bonds and molecular volume (r = 0.901, P < 0.001), number of H acceptors and H donors (r = 0.949, P < 0.001), number of H acceptors and PSA (r = 0.998, P < 0.001), number of H donors and PSA (r = 0.955, P < 0.001). For correlated variables, only one of the baseline variables was chosen to be included in the predictive model and were M_r_, PSA and log P.

**Table 1:** Pearson correlations between physicochemical properties of phytochemicals in the training dataset (N = 67).

Physico-chemical properties	M_r_	Log P	Freely rotatable bonds	H acceptors	H donors	PSA
Log P	r = 0.174					
	P = 0.159					
Freely rotatable bonds	**r = 0.772**	r = 0.554				
	**P < 0.001**	P < 0.001				
H acceptors	r = 0.442	**−0.755**	r = −0.094			
	P < 0.001	**P < 0.001**	P = 0.449			
H donors	r = 0.424	r = −0.712	r = −0.127	**r = 0.949**		
	P < 0.001	P < 0.001	P = 0.306	**P < 0.001**		
PSA	0.435	r = −0.748	r = −0.110	**r = 0.998**	**r = 0.955**	
	P < 0.001	P < 0.001	P = 0.377	**P < 0.001**	**P < 0.001**	
Molecular volume	**r = 0.949**	r = 0.445	**r = 0.901**	r = 0.413	r = 0.141	r = 0.135
	**P < 0.001**	P < 0.001	**P < 0.001**	P = 247	P = 0.254	P = 0.277

Data reported as Pearson’s correlation coefficient (r) with P-values. Significantly high correlations (|r| > 0.75, P < 0.05) are highlighted with bold.

To test the effects of dietary intake forms, Pearson correlation analyses between T_max_, M_r_, PSA and log P were performed with the inclusion of dietary intake forms (liquid, semi-solid and solid). Table 2 shows significantly high correlations between PSA and log P in the liquid intake form (r = −0.82, P < 0.001) and in the semi-solid intake form (r = −0.93, P < 0.001). Therefore, the predictive model of T_max_ was developed including 2 separate models: the ‘log P model’ containing log P and M_r_ and the ‘PSA model’ containing PSA and M_r_.

**Table 2:** Pearson correlations between selected physicochemical properties and T_max_ in the training dataset (N = 67).

Dietary intake form	Variable	T_max_	M_r_	Log P
Liquid	M_r_	r = 0.48		
		P = 0.01		
	Log P	**r = 0.80**	r = 0.45	
		**P < 0.001**	P = 0.015	
	PSA	r = −0.72	r = −0.03	**r = −0.82**
		P < 0.001	P = 0.88	**P < 0.001**
Semi-solid	M_r_	r = 0.28		
		P = 0.388		
	Log P	**r = 0.75**	r = −0.07	
		**P = 0.005**	P = 0.832	
	PSA	r = −0.64	r = 0.30	**r = −0.93**
		P = 0.026	P = 0.342	**P < 0.001**
Solid	M_r_	r = 0.52		
		P < 0.001		
	Log P	r = 0.65	r = 0.19	
		P < 0.001	P = 0.332	
	PSA	r = −0.13	r = 0.66	r = −0.56
		P = 0.517	P < 0.001	P = 0.002

Data reported as Pearson’s correlation coefficient (r) with P-values. Significantly high correlations (|r| > 0.75, P < 0.05) are highlighted with bold.

### Development of the predictive model

**Figure 1:**
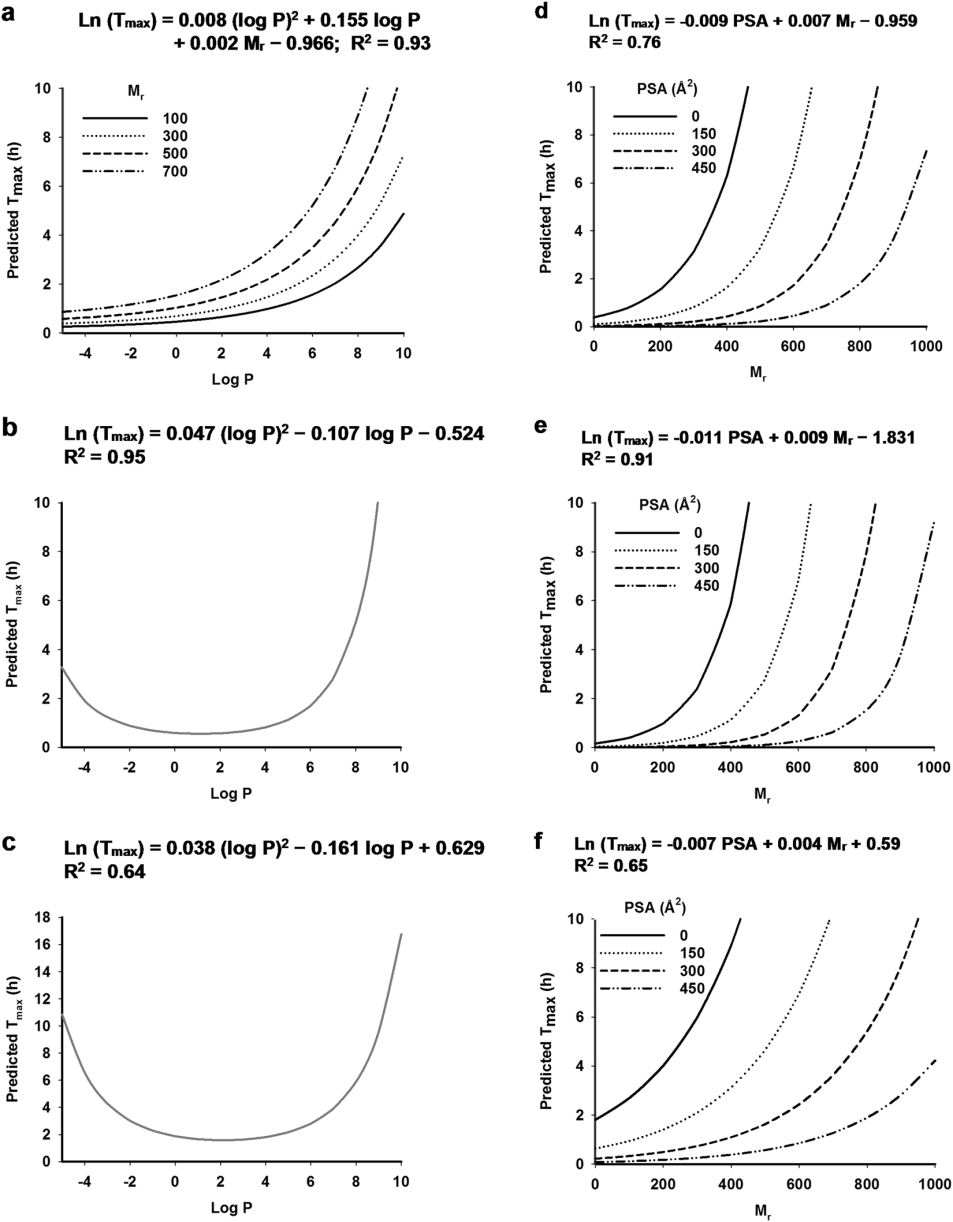
Prediction of T_max_ by the predictive model. (**a**) The log P model in liquid, (**b**) semi-solid and (**c**) solid intakes. (**d**) The PSA model in liquid, (**e**) semi-solid and (**f**) solid intakes.

To develop the predictive model of T_max_ for phytochemicals, we used regression modelling with a natural logarithm transformation of T_max_ (ln (T_max_)) and standard error (SE) of T_max_ as weights to account for the uncertainty of each data point. We used the training dataset containing 67 phytochemicals collected from 31 clinical studies with a total number of 384 healthy participants (Table 3). The predictive model included 2 mathematical models: the log P model and the PSA model that appeared to approximately equally well fit the data with coefficients depending on dietary intake forms (Fig. 1). All models had statistical power of >0.999.

**Table 3:** Summary of datasets for development and validation of the predictive model.

Dataset	Intake	No. of studies	No. of people	No. of PCs	T_max_ range^a^ (h)	M_r_ range^b^	Log P range^b^	PSA range^b^ (Å^2^)
Training	Liquid	12	112	28	0.3–32.6	122–612	−4.4–10	0–271
	Semi-solid	5	29	12	0.6–14.8	302–728	−4.7–9.8	0–309
	Solid	16	257	27	0.7–15.2	122–1270	−3.4–9.4	29–465
	Total	31	384	67	0.3–32.6	122–1270	−4.7–9.8	0–465
PCv	Liquid	19	667	70	0.5–19	138–659	−4.3–10	0–271
	Semi-solid	5	129	12	1.0–4.0	176–758	−4.7–−1.4	107–330
	Solid	14	354	26	0.8–37	176–569	−2.8–9.8	0–197
	Total	34	1150	108	0.5–37	138–758	−4.7–10	0–330
PHv-fasted	Solid	59	963	60	0.8–3.6	123–552	−1.7–5.2	3–146
PHv-fed	Solid	37	617	38	1.4–6.5	123–823	−1.7–5.4	3–221

^a^T_max_ of phytochemicals were collected from clinical studies in the literature.

^b^Physicochemical properties of phytochemicals including M_r_, Log P and PSA were calculated using the Molinspiration Chemoinformatics calculator.

**Figure 2:**
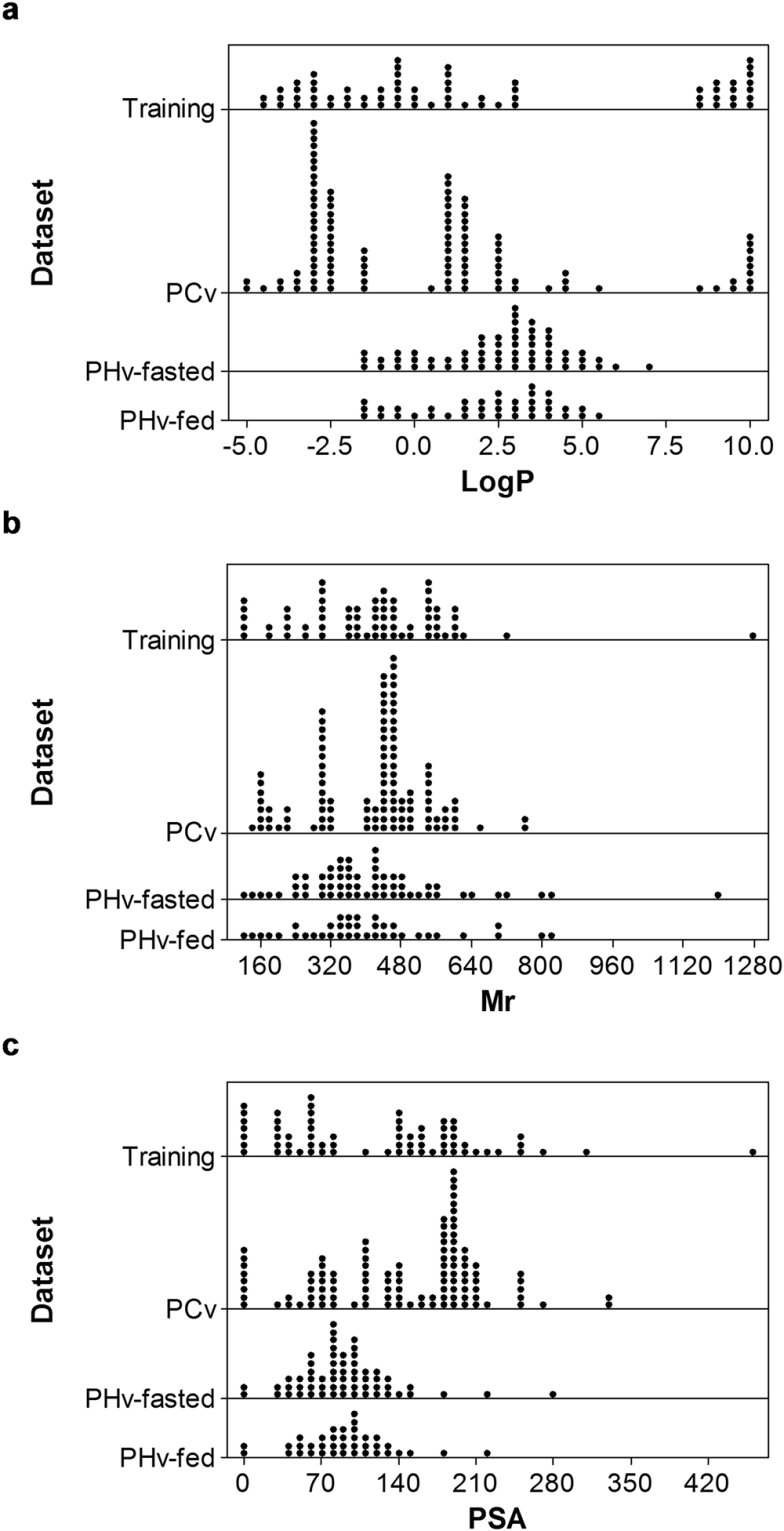
Summary of variables included in datasets for the development and validation of the predictive model. Dot plots demonstrate distributions of (**a**) log P, (**b**) M_r_ and (**c**) PSA of four datasets: training (N = 67), PCv (N = 108), PHv-fasted (N = 60) and PHv-fed (N = 38) datasets.

The log P model estimated T_max_ based on log P and M_r_ (Fig. 1a–c). When phytochemicals were administered in liquid form, ln (T_max_) was positively associated with log P and M_r_ (Fig. 1a). When phytochemicals were administered in semi-solid (Fig. 1b) or solid (Fig. 1c) forms, ln (T_max_) was independent of M_r_ and followed a quadratic relationship with log P. The PSA model estimated T_max_ based on PSA and M_r_ (Fig. 1d–f). In the PSA model, ln (T_max_) was positively associated with M_r_ and negatively associated with PSA. Overall, the predictive model covered a M_r_ range of 122–1270, a log P range of −4.7–9.8 and a PSA range of 0–465 Å^2^ corresponding a T_max_ range of 0.3–32.6 h (Table 3). Distribution patterns of log P, M_r_ and PSA in the training dataset were demonstrated in Fig. 2. Log P was relatively evenly distributed across the range from −4.7–3 and 8.7–10 (Fig. 2a). Therefore, the log P model had to interpolate values between 3 and 8.5 because they were not represented in the training dataset. M_r_ and PSA of the training dataset were evenly distributed (Fig. 2b and c).

**Figure 3:**
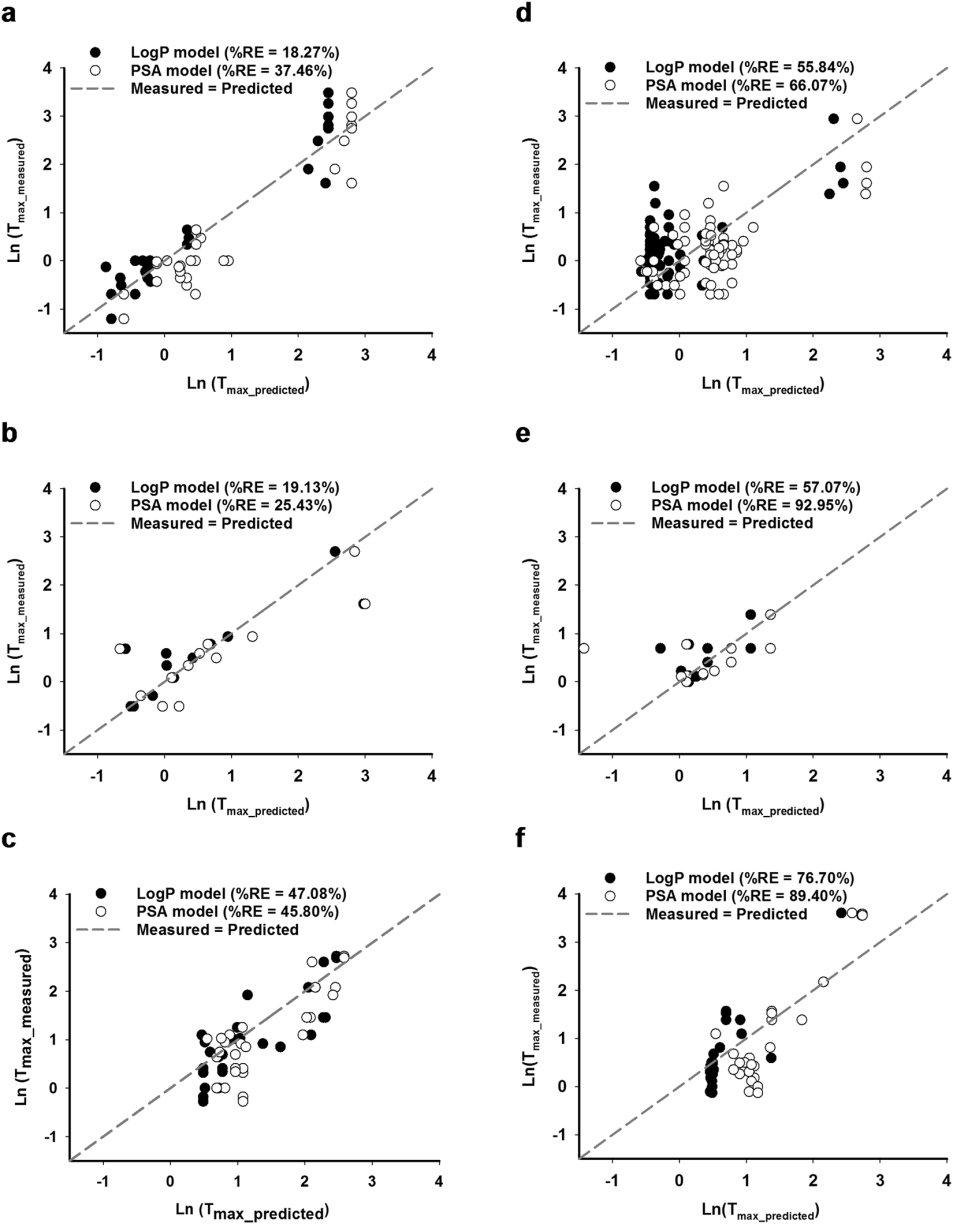
Comparison of measured versus predicted values of T_max_ of the training dataset and the PCv dataset. Natural logarithm of T_max_ measured from the training dataset (N = 67) were plotted against natural logarithm of predicted T_max_ based on the log P model (black circle), the PSA model (clear circle) and compared to the regression of measured T_max_ = predicted T_max_ (dotted line) when intake as (**a**) liquid, (**b**) semi-solid and (**c**) solid forms. Similar comparison was plotted for the PCv dataset (N = 108) when intake as (**d**) liquid, (**e**) semi-solid and (**f**) solid forms.

The prediction accuracy of the log P model and the PSA model in the training dataset was assessed by the root mean weighted square error normalized by the weights (RNMSWE) and the percentage relative error (%RE) of predictions (Table 4). Comparison of the measured versus predicted values of ln (T_max_) was plotted in Fig. 3a–c. The RNMSWE of prediction is an estimate of the standard deviation of the prediction normalized by the weights. As T_max_ required a natural logarithm transformation, the RNMSWE in ln (hours) was transformed to %RE of prediction which is approximately average % error of T_max_ (in hours) over the mean of T_max_ (in hours). The %RE of prediction of the log P model was 18.27%, 19.13% and 47.08% for the liquid, semi-solid and solid intakes, respectively. The %RE of prediction of the PSA model was 37.46%, 25.43% and 45.8% for the liquid, semi-solid and solid intakes, respectively (Table 4). Overall, for the training dataset, despite the similar R^2^, the log P model had lower %RE of prediction across all three intakes and thus higher prediction accuracy.

**Table 4:** Comparison of prediction accuracy of the predictive model for each dataset.

Parameter	Log P model				PSA model			
	Training dataset	PCv dataset	PHv-fasted	PHv-fed	Training dataset	PCv dataset	PHv-fasted	PHv-fed
*Liquid intake*								
NMSWE	0.0282	0.1968	NA	NA	0.1012	0.2573	NA	NA
RNMSWE	0.1678	0.4436	NA	NA	0.3181	0.5072	NA	NA
%RE	18.27	55.84	NA	NA	37.46	66.07	NA	NA
N	28	70	NA	NA	28	70	NA	NA
*Semi-solid intake*								
NMSWE	0.0306	0.2039	NA	NA	0.0513	0.4320	NA	NA
RNMSWE	0.1750	0.4516	NA	NA	0.2266	0.6573	NA	NA
%RE	19.13	57.07	NA	NA	25.43	92.95	NA	NA
N	12	12	NA	NA	12	12	NA	NA
*Solid intake*								
NMSWE	0.1488	0.3241	0.1390	0.4349	0.1422	0.4079	0.9327	0.4256
RNMSWE	0.3858	0.5693	0.3728	0.6594	0.3771	0.6387	0.9658	0.6524
%RE	47.08	76.70	45.18	93.37	45.80	89.40	162.69	92.01
N	27	26	60	38	27	26	60	38

### Validation of the predictive model

To validate the predictive model, we used three independent datasets: the PCv, PHv-fasted and PHv-fed datasets. In comparison with the training dataset, all three validation datasets covered smaller ranges of log P, M_r_ and PSA (Table 3, Fig. 2). The PCv dataset contained phytochemicals of similar chemical classes to the training dataset whilst the PHv-fasted and the PHv-fed datasets contains pharmaceutical compounds. The PCv dataset contained 108 phytochemicals including anthocyanins, flavanols, flavonols, hydrobenzoic acids, hydroxycinnamic acids, stilbenes, carotenoids and vitamins (Supplementary Table S2). Comparing to the training dataset, the PCv dataset covered a similar range of log P of −4.7–10 and measured T_max_ of 0.5–37 h (Table 3) with sparsely distributed data of log P (Fig. 2a). Log P values of the PCv dataset were more concentrated in the range of −2.8–−2.5 and 1.2–2.3. Similar to the training dataset, the PCv dataset lacked log P values from 5.6–8.4 (Fig. 2a). The PCv dataset covered a M_r_ range of 138–758 and a PSA range of 0–330 Å^2^ (Table 3, Fig. 2b and c). In comparison the training dataset, M_r_ and PSA of the PCv dataset were less evenly distributed (Fig. 2b and c).

To evaluate the prediction accuracy of the predictive model on the PCv dataset, we compared the measured versus predicted values of ln (T_max_) in Fig. 3d–f and calculated the %RE in Table 4. The %RE of prediction of the log P model was 55.84%, 57.07% and 76.7% for the liquid, semi-solid and solid intakes, respectively. The %RE of prediction of the PSA model was 66.07%, 92.95% and 89.4% for the liquid, semi-solid and solid intakes, respectively (Table 4). Overall, for the PCv dataset and in comparison with the PSA model, the log P model had lower %RE of prediction across three intakes and thus higher prediction accuracy. Comparing to the training dataset, the PCv dataset had higher %RE of prediction and thus lower prediction accuracy across all intake forms.

To validate the predictive model on pharmaceutical compounds, we used two pharmaceuticals validation datasets: PHv-fasted and PHv-fed. All pharmaceutical compounds in the two datasets were administered in the solid form (Table 3). The PHv-fasted dataset contains 60 compounds collected from 59 clinical studies and the PHv-fed dataset contains 38 compounds collected from 37 clinical studies (Table 3). The entire list of pharmaceutical compounds in the PHv-fasted dataset can be found as Supplementary Table S3 and the PHv-fed dataset as Supplementary Table S4. The two PHv datasets covered a similar range of log P −1.7–5.4 (Table 3) with a similar distribution pattern (Fig. 2a). Comparing to the PHv-fasted dataset, the PHv-fed dataset covered a slightly broader range of M_r_ of 123–823 and PSA of 3–221 Å^2^ while the PHv-fasted dataset covered M_r_ range of 123–552 and PSA of 3–146 Å^2^ (Table 3). Similar distribution patterns of M_r_ and PSA were observed in the two PHv datasets (Fig. 2b and c).

**Figure 4:**
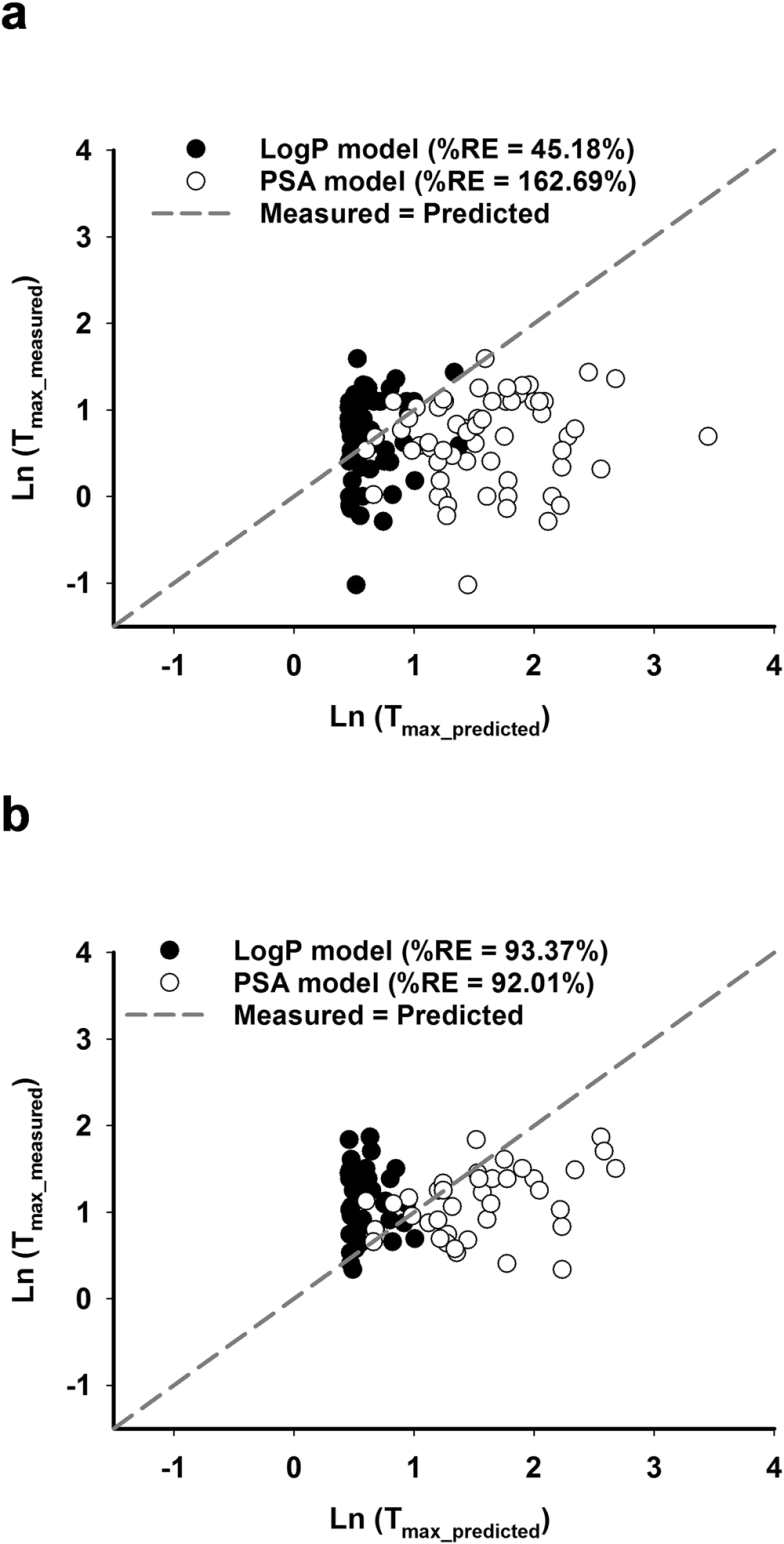
Comparison of measured versus predicted values of T_max_ of the PHv datasets. Natural logarithm of T_max_ measured from (**a**) the PHv-fasted (N = 60) dataset and (**b**) the PHv-fed (N = 38) dataset were plotted against natural logarithm of predicted T_max_ based on the log P model (black circle), the PSA model (clear circle) and compared to the regression of measured T_max_ = predicted T_max_ (dotted line) when intake as solid forms.

To evaluate the effects of food on the prediction accuracy of the model, we compared the measured versus predicted values of ln (T_max_) in Fig. 4 and calculated the %RE in Table 4. The %RE of prediction for the log P model was 45.18% for the PHv-fasted dataset and 93.37% for the PHv-fed dataset. The %RE of prediction for the PSA model was 162.69% for the PHv-fasted dataset and 92.01% for the PHv-fed dataset (Table 4). For the log P model, food increased the %RE of prediction and therefore reduced the prediction accuracy. By contrast, for the PSA model, food reduced the %RE of prediction and thus increased the prediction accuracy. Overall, the log P model and PSA model had similar %RE for the PHv-fed dataset. However, the log P model had substantially lower %RE for the PHv-fasted dataset and thus had higher prediction accuracy.

## Discussion

This is the first direct model to predict the time of maximal plasma concentration (T_max_) of dietary phytochemicals in the human body based on their physicochemical properties and dietary intake forms. The model was developed based on T_max_ data from clinical studies of healthy individuals and therefore predicts the absorption of phytochemicals in the human body. To select the most important variables for the predictive model, we analysed the correlation between several physicochemical properties that are well known in pharmaceutical science to have significant impacts on oral bioavailability of drugs such as molecular mass, lipophilicity, polar surface area, molecular volume, number of freely rotatable bonds, number of hydrogen donors and acceptors^28–30^. We found significantly high correlation between some of the physicochemical properties and selected three independent physicochemical properties to use in the model including molecular mass, lipophilicity and polar surface area. These phytochemical properties were selected due to their well-known impacts on drug bioavailability as they are related to intestinal membrane permeability of a compound^28, 29^. In order for a drug to cross the membrane, the compound needs to break hydrogen bonds with its aqueous environment and partition through the membrane^152^. Polar surface area is related to the hydrogen-bonding potential of a compound whilst molecular mass and lipophilicity are related to the membrane permeability. Consistent with the literature^28, 29, 152^, we found that these physicochemical properties had significant impacts on the T_max_ of dietary phytochemicals in the human body. Further, dietary intake forms were ﻿also identified to have a significant impact on absorption of dietary phytochemicals and were included in the model development. Similar to drug compounds, the effects of dietary intake forms on bioavailability of phytochemicals are related to the dissolution of phytochemicals within the gastrointestinal tract making them available for absorption^153^. Therefore, comparing to the liquid form, dietary phytochemicals consumed in the semi-solid or solid forms would require longer time to dissolve into the gastrointestinal environment before they are available for absorption.

The predictive model based on lipophilicity and molecular mass provides a quantitative and high-throughput tool for prediction of T_max_ of dietary phytochemicals and also pharmaceutical compounds ingested without food. T_max_ of a phytochemical or pharmaceutical compound that has not been studied *in vivo* can thereby be calculated from its molecular mass and log P for three different intake forms of liquid, semi-solid or solids using the equations reported in this predictive model (Fig. 1a–c). For example, phytochemical phloretin (M_r_ = 274.27, log P = 2.66) found in apple would be predicted to have T_max_ of 1.05, 0.62 and 1.6 h when consumed in liquid, semi-solid and solid forms, respectively. The model covers a broad range of chemical classes from phenolic compounds to carotenoids, from very hydrophilic (log P ~ −4.7) to very lipophilic (log P ~ 10) with a wide molecular mass range of M_r_ ~ 122–1270. The prediction accuracy of the model was indicated by relative error of prediction from 18–77% for total 175 dietary phytochemicals tested and 45% for 60 pharmaceutical compounds ingested without food (Table 4). The relative error of prediction is an indication of the total error of prediction compared to the mean. Our literature searches show that published T_max_ have a SE between 0 and 200% of the mean (Supplementary Tables S1–S4). Therefore, the prediction accuracy of our model was deemed adequately accurate for valid prediction of T_max_. Additionally, considering that a statistical power of 0.8 is the standard for adequacy^154^, our model with power of >0.999 had high statistical power for confidence in preduction accuracy.

The predictive model was of course limited by the literature reports of the experimental data. The T_max_ variable was logarithmically transformed to alleviate the non-normality of the errors. However, there were gaps in the independent variables of log P from 3–8.5 and M_r_ from 750–1270 that the model had to overcome (Fig. 2). Therefore, further data covering a complete range of the parameter space would increase the rigour of the model. Additionally, we observed an increase of relative error of prediction for pharmaceutical compounds when ingested with food (Table 4). Mechanisms whereby food affects the bioavailability of drug absorption have been well studied. Food promotes absorption of lipophilic drugs due to improved drug solubilisation whilst reducing absorption of hydrophilic drugs due to delayed drug permeation^155^. Similar effects of food on absorption of dietary phytochemicals have been observed^20^. Increased absorption of the lipophilic compound lycopene in tomato was reported when consumed with olive oil^156^. Hydrophilic compounds such as phenolic acids and anthocyanins were observed to bind to fibre and compromised their absorption during stimulated gastric and small intestinal digestion^157^. Further, protein in food has been reported to reduce absorption of dietary phytochemicals in chocolate^158^. Our predictive model was developed based on dietary phytochemicals administered as single-source phytochemicals or phytochemical extracts and also phytochemicals consumed in their natural matrices of whole fruits and vegetables (Supplementary Table S1). Apart from the models for phytochemicals consumed in liquid (Fig. 1a) or solid (Fig. 1c) forms, mostly in isolation or extracts, a statistically valid model was also developed from consumption of phytochemicals mostly (75%) in whole fruits and vegetables and accounted for the effects of these matrices on phytochemical absorption in semi-solid form (Fig. 1b). Therefore, the effects of interactions of phytochemicals with macronutrients such as fibre and protein from the natural matrices were accounted for to a small extent. Accordingly, Conversely, the impact of macronutrients from food sources other than natural plant food matrices on T_max_ of phytochemicals are not accounted for. Considering that macronutrients are known to interact with phytochemicals and thereby alter their T_max_^20^, the developed model may less accurately predict the T_max_ of phytochemicals when consumed in conjunction with other foods. Accordingly, the predictive model reported herein is most applicable for prediction of T_max_ of dietary phytochemicals and pharmaceuticals ingested without foods.

In this study, the time of maximal plasma concentration (T_max_) was chosen as the most relevant molecular data for the predictive model due to its importance in understanding and optimising the health benefits of dietary phytochemicals. Phytochemicals are treated as xenobiotic species and therefore display transient presence in circulation^16^. Under this circumstance, the T_max_ is of prime importance in predicting the presence of any phytochemicals with the expectation that it will be substantially eliminated after a few hours or a few days depending on the phytochemicals^18, 19^. The protective efficacy of dietary phytochemicals can mitigate oxidative stress and inflammation (OSI) associated with daily activity and found consistently elevated in chronic diseases^7–9^. Managing OSI associated with daily activity is likely an important strategy for reducing disease risk in both healthy and unhealthy people. The time of maximal plasma concentration of dietary phytochemicals has recently been reported to have an important impact on their ability to regulate OSI^159^. Consumption of a strawberry drink 2 h before a high fat meal maximises protection against OSI compared with having the drink with or 2 h after the meal^159^, supporting that the T_max_ of dietary phytochemicals must be matched to the OSI challenge for optimal health protection^159^. The T_max_ of strawberry phytochemicals were reported to be about 1–2 h therefore consumption of the strawberry drink 2 h before the meal allowed their presence at maximal plasma concentration to reduce the OSI burden stimulated by the high fat meal^160^. Here, we chose T_max_ instead of maximal plasma concentration (C_max_) in the predictive model as T_max_ seems to be less affected by dose. For example, T_max_ of lycopene was reported to be about 5 h irrespective of the dose whilst C_max_ increased with dose escalation^65^. Furthermore, the anti-OSI response of phytochemicals does not necessarily continue to increase with dose and higher concentrations of phytochemicals may become pro-oxidants and promote OSI^161–163^. Without good understanding of the target C_max_ for maximising phytochemical efficacy, C_max_ is less useful than T_max_.

Although the study is not concerned with post-primary absorption of phytochemicals formed during hepatic and microbial metabolism, it is acknowledged that these metabolites may also contribute to the regulation of OSI similarly to their parent compounds^164–166^. Therefore, it is important to consider the reported T_max_ of these derived metabolites (not predicted by the model) together with T_max_ of the parent compounds predicted by this model. The main hepatic metabolites of phytochemicals are glucuronide, sulphate and methylation derivatives with short T_max_ values that range from 0.5 h to up to 2.5 h^42^, indicative of rapid clearance by the hepatic portal system. Colonic microbiota chemical transformations of phytochemicals include hydrolysation, reduction, ring-cleavage, demethylation and dihydroxylation of both parent compounds and their hepatic derivatives^167, 168^. Accordingly, metabolites with T_max_ > 5 h are likely to be absorbed or transformed with the involvement of the colonic microbiota^169^.

The ability to predict T_max_ of dietary phytochemicals offers a valuable tool for designing clinical studies to capture the time of maximal phytochemicals in the human body and to avoid underestimation of their impacts on regulation of OSI. We propose that by matching T_max_ to the biological cycle of OSI, suppression of OSI is maximised and the associated tissue damage would be minimised. Therefore, the strategy for optimising the protective efficacy of dietary phytochemicals involves selection of phytochemical sources to achieve desirable T_max_ that target different needs for OSI regulation. Using the unique approach of combining phytochemical-rich foods based on computable physicochemical properties, we can understand the absorption characteristics of dietary phytochemicals to achieve their full potential for protective health benefits.

## Methods

### Clinical data collection

Clinical measures of T_max_ were obtained from the literature using the PubMed database. Information collected included compound name and family, sources, dose, intake forms and T_max_ in hours (as mean ± SE, hours). When T_max_ was given as median and range, conversion to mean and SE was performed as described in Hozo *et al*.^170^. The inclusion selection criteria for publications included: 1) randomised controlled clinical trials in healthy volunteers; 2) inclusion of a wash-out period when the study followed a cross over design; 3) PCs analysed were passively absorbed, i.e., compounds found in the plasma or serum were unchanged from those ingested; and 4) plasma analysed without enzymatic deconjugation.

The data collected here were included in the training dataset.

### Physicochemical property data collection

Physicochemical properties of phytochemicals were calculated from the molecular structures using the Molinspiration Chemoinformatics calculator (www.molinspiration.com). The physicochemical properties calculated included M_r_, log P, PSA, number of freely rotatable bonds, number of H acceptors, number of H donors and molecular volume.

### Pearson correlation analysis between variables in the training dataset

Pearson correlation analyses of all variables included in the training dataset were performed using the statistical package R version 3.3.2^171^. Results were reported as Pearson’s correlation coefficient (r) and P-values.

### Development of the predictive model

The predictive model was developed by a linear model framework using the statistical package R. The dependent variable T_max_ required a natural logarithm transformation (ln(T_max_)) to capture the non-normality of errors in the variance across all observations of T_max_. The SE of each sample was used as weights during the regression modelling of T_max_. Because T_max_ required a log normal distribution, and since:

1 $$Var(ln(Y))\approx \frac{S{E}^{2}(Y)}{{E}^{2}(Y)},$$

where E(Y) = expected value of y = mean(y)

the calculated weights for the regression modelling were:

2 $$w=1/{(SE({T}_{max})/{T}_{max})}^{2}$$

when SE was missing, the weight was set to 4 and when SE was zero the weight was set to 400. Significance testing between T_max_ and the physicochemical properties of phytochemicals was carried out using multivariate regression.

### Power analysis of the predictive model

Post hoc power analysis of the predictive model was performed using the power calculation program G*Power 3.1.9.2^172, 173^.

### Validation of the predictive model

The prediction accuracy of the predictive model was validated using three independent datasets of measured T_max_ obtained from clinical studies using the same selection criteria, including the PCv, PHv-fasted and PHv-fed datasets. Measured T_max_ was collected as mean ± SE (hours). The prediction accuracy of the predictive model was evaluated by the normalised mean square weighted error (NMSWE) and % relative error of prediction for each dataset. The NMSWE of prediction was calculated:

3 $$NMSWE(\hat{Y})=\frac{{\sum }_{1}^{N}{w}_{i}{({Y}_{i}-{\hat{Y}}_{i})}^{2}}{{\sum }_{1}^{N}{w}_{i}}$$

where w_i_ is the weights calculated as in Equation 1, Y_i_ is ln(T_max_measured_), Ŷ_i_ is ln(T_max_predicted_) and N is the number of data points.

Root NMSWE (RNMSWE) was calculated:

4 $$RNMSWE=\sqrt{NMSWE}$$

Let Δ=RNMSWE of prediction. If ɛ is the error in predicted values of T_max_ and ln(T_max_ + ɛ) is predicted from the predictive model, then:

5 $${\rm{\Delta }}\approx \,\mathrm{ln}({T}_{max}+\varepsilon )-\,\mathrm{ln}({T}_{max})\approx \,\mathrm{ln}(\frac{{T}_{max}+\varepsilon }{{T}_{max}})\approx ln(1+\frac{\varepsilon }{{T}_{max}})$$

Converting Δ (ln hours) to hours:

6 $${e}^{{\rm{\Delta }}}=1+\frac{\varepsilon }{{T}_{max}}$$

The % relative error (RE) of prediction is an approximately averaged error over all data points in the dataset:

7 $$ \% RE=\frac{\varepsilon }{{T}_{max}}\,\times 100=({e}^{{\rm{\Delta }}}-1)\times 100$$

## Electronic supplementary material

Supplementary Information
