# Supplementary material for: Physicochemical properties of dietary phytochemicals can predict their passive absorption in the human small intestine
Source: Sci Rep. 2017 May 16;7:1931. doi: 10.1038/s41598-017-01888-w (PMC5434065; doi:10.1038/s41598-017-01888-w)
Supplement: Supplementary file 1 — Supplementary Information [file 41598_2017_1888_MOESM1_ESM.pdf]

Physicochemical properties of dietary phytochemicals can predict their passive absorption in the human small intestine

Sophie N.B. Selby-Pham[1,2], Rosalind B. Miller[3], Kate Howell[1], Frank Dunshea[1] and Louise E. Bennett[2,*]

[1]Faculty of Veterinary and Agricultural Science, The University of Melbourne, Parkville, 3010, Australia

[2]CSIRO Agriculture and Food, 671 Sneydes Road, Werribee, 3030, Australia

[3]CSIRO Data61, North Ryde, 2113, Australia

[*]Louise.Bennett@csiro.au

S1 Table. The training dataset used for the development of the predictive model.

**Phytochemical**	**M_r***	**LogP***	**PSA* (Å^2)**	**No. freely rotatable bonds***	**No. H acceptor***	**No. H donor***	**Molecular volume* (Å^3)**	**Intake#**	**T_{max}# (h) (mean ± SE)**	**Reference #**
Anthocyanins										
Cyanidin 3-O-arabinoside	419.36	-1.627	171.234	4	10	7	342.085	solid (extract)	3.5 ± 0.5	31
Cyanidin 3-O-galactoside	449.38	-2.793	191.462	4	11	8	366.931	solid (extract)	2.5 ± 0.5	31
Cyanidin 3-O-glucoside	449.38	-2.793	191.462	4	11	8	366.931	liquid (juice)	0.5	54
Cyanidin 3-O-glucoside	449.38	-2.793	191.462	4	11	8	366.931	semi-solid (freeze-dried whole fruit made into a paste)	1.09 ± 0.3	52
Cyanidin 3-O-glucoside	449.38	-2.793	191.462	4	11	8	366.931	liquid (juice)	1	49
Cyanidin 3-O-rutinoside	595.53	-3.492	250.386	6	15	10	490.793	semi-solid (freeze-dried whole fruit made into a paste)	1.64 ± 0.5	52
Cyanidin 3-O-rutinoside	595.53	-3.492	250.386	6	15	10	490.793	liquid (juice)	1	49
Cyanidin 3-O-sambubioside	581.50	-4.071	250.386	6	15	10	474.206	semi-solid (freeze-dried whole fruit made into a paste)	2.18 ± 1.54	52
Cyanidin 3-O-xylosyl-rutinoside	727.65	-4.572	309.31	8	19	12	598.069	semi-solid (freeze-dried whole fruit made into a paste)	2.55 ± 0.169	52

Delphinidin 3-O-glucoside	465.39	-3.084	211.69	4	12	9	374.948	liquid (juice)	1	49
Delphinidin 3-O-rutinoside	611.53	-3.783	270.614	6	16	11	498.811	liquid (juice)	1	49
Malvidin 3-O-glucoside	493.44	-2.469	189.702	6	12	7	410.004	semi-solid (extract with yoghurt)	1.8 ± 0.6	36
Peonidin 3-O-glucoside	463.42	-2.486	180.468	5	11	7	384.458	semi-solid (extract with yoghurt)	1.4 ± 0.8	36
Flavanols										
(−)-Epicatechin 3-O-gallate	442.37	2.537	177.135	4	10	7	359.554	liquid (drink)	1.6 ± 0.2	18
(−)-Epigallocatechin 3-O-gallate	458.37	2.245	197.363	4	11	8	367.571	liquid (drink)	1.9 ± 0.1	18
(−)-Epigallocatechin 3-O-gallate	458.37	2.245	197.363	4	11	8	367.571	liquid (drink)	1.4 ± 0.6	34
Flavonols										
Isorhamnetin 3-O-glucuronide	492.39	-0.179	216.58	5	13	7	391.915	semi-solid (fried red onions)	0.6 ± 0.1	42
Quercetin aglycone	302.24	1.683	131.351	1	7	5	240.084	solid (pure compound)	3 ± 2	41
Quercetin 3-O-glucuronide	478.36	-0.487	227.574	4	13	8	374.387	semi-solid (fried red onions)	0.6 ± 0.1	42
Quercetin 3'-O-sulfate	381.29	-1.809	177.556	3	10	4	277.758	semi-solid (fried red onions)	0.75 ± 0.12	42
Hydroxybenzoic acids										

Ellagic acid	302.19	0.943	141.334	0	8	4	221.776	liquid (juice)	0.65 ± 0.23	21
Ellagic acid	302.19	0.943	141.334	0	8	4	221.776	liquid (extract drink)	0.94 ± 0.06	21
Ellagic acid	302.19	0.943	141.334	0	8	4	221.776	solid (extract)	2.58 ± 0.42	21
Ellagic acid	302.19	0.943	141.334	0	8	4	221.776	semi-solid (freeze-dried whole fruit made into a paste)	1.98 ± 2.87	52
Ellagic acid	302.19	0.943	141.334	0	8	4	221.776	solid (extract)	1	40
Ellagic acid	302.19	0.943	141.334	0	8	4	221.776	liquid (juice)	0.98 ± 0.06	50
Hydroxycinnamic acids										
5-O-Caffeoylquinic acid	354.31	-0.453	164.744	5	9	6	289.267	liquid (drink)	1 ± 0.2	51
3-O-caffeoylquinic acid lactone-O-sulfate	415.35	-4.212	179.727	6	11	3	316.048	liquid (drink)	0.6 ± 0.1	51
4-O-caffeoylquinic acid lactone-O-sulfate	415.35	-4.375	179.727	6	11	3	316.048	liquid (drink)	0.7 ± 0.1	51
3-O-Feruloylquinic acid	368.34	-0.145	153.75	6	9	5	313.795	liquid (drink)	0.7 ± 0.1	51

4-O-Feruloylquinic acid	368.34	-0.363	153.75	6	9	5	313.795	liquid (drink)	0.8 ± 0.1	51
5-O-Feruloylquinic acid	368.34	-0.145	153.75	6	9	5	313.795	liquid (drink)	0.9 ± 0.1	51
Stilbenes										
trans-Resveratrol	228.24	2.986	60.684	2	3	3	206.922	solid (pure compound)	0.833	32
trans-Resveratrol	228.24	2.986	60.684	2	3	3	206.922	solid (pure compound)	0.759	32
trans-Resveratrol	228.24	2.986	60.684	2	3	3	206.922	solid (pure compound)	1.375	32
trans-Resveratrol	228.24	2.986	60.684	2	3	3	206.922	solid (pure compound)	1.5	32
Carotenoids										
Lycopene	536.89	9.977	0	16	0	0	601.871	liquid (drink)	16.6 ± 10.1	19
Lycopene	536.89	9.977	0	16	0	0	601.871	liquid (drink)	19.8 ± 12.4	19
Lycopene	536.89	9.977	0	16	0	0	601.871	liquid (drink)	15.6 ± 13.8	19
Lycopene	536.89	9.977	0	16	0	0	601.871	liquid (drink)	26.1 ± 5.5	19
Lycopene	536.89	9.977	0	16	0	0	601.871	liquid (drink)	32.6 ± 18.6	19
β-Carotene	536.89	9.843	0	10	0	0	591.964	semi-solid (pure compound in emulsion)	5	47
β-Carotene	536.89	9.843	0	10	0	0	591.964	liquid (drink)	5	46

Astaxanthin	596.85	8.596	74.598	10	4	2	612.415	solid (pure compound)	8 ± 0	44
Astaxanthin	596.85	8.596	74.598	10	4	2	612.415	liquid (drink)	6.7 ± 1.2	45
Canthaxanthin	564.85	9.293	34.142	10	2	0	596.328	liquid (drink)	12	46
all-E-Zeaxanthin	568.89	9.39	40.456	10	2	2	608.052	solid (pure compound)	15.2 ± 7.6	37
all-E-Zeaxanthin	568.89	9.39	40.456	10	2	2	608.052	solid (pure compound)	14.7 ± 0.9	37
Lutein	568.89	9.307	40.456	10	2	2	608.077	semi-solid (pure compound)	14.8 ± 2.5	55
Vitamins										
Vitamin C (ascorbic acid)	176.12	-1.402	107.22	2	6	4	139.71	solid (pure compound)	2.76 ± 0.62	24
Vitamin B1 (thiamine)	265.36	-3.449	75.92	4	5	3	239.76	liquid (pure compound)	0.88	53
Vitamin B1 (thiamine)	265.36	-3.449	75.92	4	5	3	239.76	solid (pure compound)	2.34 ± 0.89	39
Vitamin B2 (riboflavin)	376.40	-0.759	161.563	5	10	5	321.314	solid (pure compound)	1.5	57
Vitamin B2 (riboflavin)	376.40	-0.759	161.563	5	10	5	321.314	solid (pure compound)	1.4	57
Vitamin B2 (riboflavin)	376.40	-0.759	161.563	5	10	5	321.314	solid (pure compound)	2	57
Nicotiamide	122.13	-0.48	55.986	1	3	2	110.159	liquid (pure compound)	0.3 ± 0.1	48
Nicotiamide	122.13	-0.48	55.986	1	3	2	110.159	liquid (pure compound)	0.5 ± 0.3	48
Nicotiamide	122.13	-0.48	55.986	1	3	2	110.159	solid (pure compound)	1 ± 0.8	48

Nicotiamide	122.13	-0.48	55.986	1	3	2	110.159	solid (pure compound)	1.9 ± 1.2	48
Nicotinic acid	123.11	0.273	50.191	1	3	1	106.888	solid (pure compound)	2.1 ± 1.6	38
Nicotinuric acid	180.16	-1.773	79.289	3	5	2	155.076	solid (pure compound)	2.8 ± 1.4	38
Cobalamin (vitamin B12)	1270.44	-2.137	464.966	26	27	15	1146.798	solid (pure compound)	6.83 ± 3.19	33
R,*R*,*R*-α-Tocopherol	430.72	9.043	29.462	12	2	1	474.499	solid (pure compound)	13.5 ± 1.4	35
α-Tocotrienol	424.67	9.089	29.462	9	2	1	455.861	solid (pure compound)	4.3 ± 0.7	56
γ-Tocotrienol	410.64	9.03	29.462	9	2	1	439.3	solid (pure compound)	4.3 ± 0.7	56
δ-Tocotrienol	396.62	8.671	29.462	9	2	1	422.739	solid (pure compound)	3 ± 0.4	56
Phylloquinone (vitamin K)	450.71	8.803	34.142	14	2	0	483.869	solid (whole vegetable)	8	43

[*] calculated using the Molinspiration Chemoinformatics calculator

[#] collected from the literature

S2 Table. The PCv dataset for validation of the predictive model.

Family	Phyto-chemical	Source[#]	LogP[*]	M_r[*]	PSA[*] (Å^2)	Predicted T_{max}_LogP[**] (h)	Predicted T_{max}_PSA[**] (h)	Measured T_{max}[#](h)			Reference[#]
								Mean	SE	N	
Liquid intake											
Anthocyanins	Cyanidin 3-O-arabinoside	Cranberry (juice)	-1.63	419.36	171.23	0.70	1.55	3.30	1.17	10	76
	Cyanidin 3-O-arabinoside	Cranberry (juice)	-1.63	419.36	171.23	0.70	1.55	1.47	0.17	15	78
	Cyanidin 3-O-galactoside	Cranberry (juice)	-2.79	449.39	191.46	0.65	1.59	1.27	0.15	15	78
	Cyanidin 3-O-galactoside	Cranberry (juice)	-2.79	449.39	191.46	0.65	1.59	2.30	1.01	10	76
	Cyanidin 3-O-glucoside	Acai berry (juice)	-2.79	449.39	191.46	0.65	1.59	2.00	0.22	12	77
	Cyanidin 3-O-glucoside	Black currant (extract)	-2.79	449.39	191.46	0.65	1.59	1.25	0.16	8	75
	Cyanidin 3-O-glucoside	Elderberry (extract)	-2.79	449.39	191.46	0.65	1.59	1.08	0.34	4	62
	Cyanidin 3-O-glucoside	Cranberry (juice)	-2.79	449.39	191.46	0.65	1.59	1.13	0.21	15	78
	Cyanidin 3-O-glucoside	Red grape (juice)	-2.79	449.39	191.46	0.65	1.59	0.50	0.07	9	66
	Cyanidin 3-O-glucoside	Red grape and blueberry	-2.79	449.39	191.46	0.65	1.59	0.92	0.12	10	73
	Cyanidin 3-O-glucoside	Cranberry (juice)	-2.79	449.39	191.46	0.65	1.59	1.70	0.19	10	76
	Cyanidin 3-O-rutinoside	Black currant (extract)	-3.49	595.53	250.39	0.80	2.60	1.50	0.19	8	75

	Cyanidin 3-O-sambubioside	Elderberry (extract)	-4.07	581.5	250.39	0.74	2.36	1.19	0.28	4	62
	Cyanidin 3-O-sambubioside	Hibiscus (extract)	-4.07	581.5	250.39	0.74	2.36	1.25	0.06	6	67
	Delphinidin 3-O-glucoside	Red grape (juice)	-3.08	465.39	211.69	0.65	1.48	1.40	0.40	8	88
	Delphinidin 3-O-glucoside	Red grape (juice)	-3.08	465.39	211.69	0.65	1.48	0.63	0.08	9	66
	Delphinidin 3-O-glucoside	Red grape and blueberry (juice)	-3.08	465.39	211.69	0.65	1.48	0.97	0.08	10	73
	Delphinidin 3-O-glucoside	Black currant (extract)	-3.08	465.39	211.69	0.65	1.48	1.50	0.19	8	75
	Delphinidin 3-O-rutinoside	Black currant (extract)	-3.08	465.39	211.69	0.81	2.43	1.75	0.37	8	75
	Delphinidin 3-O-sambubioside	Hibiscus (extract)	-4.31	597.5	270.61	0.75	2.20	1.38	0.20	6	67
	Malvidin 3-O-glucoside	Cranberry (juice)	-2.47	493.44	189.7	0.73	2.20	0.93	0.28	15	78
	Malvidin 3-O-glucoside	Red grape (juice)	-2.47	493.44	189.7	0.73	2.20	0.63	0.08	9	66
	Malvidin 3-O-glucoside	Red grape (juice)	-2.47	493.44	189.7	0.73	2.20	1.38	0.08	9	66
	Malvidin 3-O-glucoside	Red grape and blueberry (juice)	-2.47	493.44	189.7	0.73	2.20	1.13	0.13	10	73
	Peonidin 3-O-arabinoside	Cranberry (juice)	-1.32	433.39	160.24	0.75	1.88	0.90	0.16	10	76
	Peonidin 3-O-arabinoside	Cranberry (juice)	-1.32	433.39	160.24	0.75	1.88	1.27	0.15	15	78
	Peonidin 3-O-galactoside	Cranberry (juice)	-2.49	463.42	180.47	0.69	1.94	1.47	0.17	15	78
	Peonidin 3-O-galactoside	Cranberry (juice)	-2.49	463.42	180.47	0.69	1.94	1.60	0.82	10	76

	Peonidin 3-O-glucoside	Red grape (juice)	-2.49	463.42	180.47	0.69	1.94	0.50	0.07	9	66
	Peonidin 3-O-glucoside	Cranberry (juice)	-2.49	463.42	180.47	0.69	1.94	1.40	0.21	15	78
	Peonidin 3-O-glucoside	Red wine (drink)	-2.49	463.42	180.47	0.69	1.94	1.38	0.08	9	66
	Peonidin 3-O-glucoside	Red grape and blueberry (juice)	-2.49	463.42	180.47	0.69	1.94	0.92	0.05	10	73
	Peonidin 3-O-glucoside	Cranberry (juice)	-2.49	463.42	180.47	0.69	1.94	4.70	3.00	10	76
	Petunidin 3-O-glucoside	Red wine (drink)	-2.78	479.41	200.7	0.69	1.94	1.25	0.05	9	66
	Petunidin 3-O-glucoside	Red grape (juice)	-2.78	479.41	200.7	0.69	1.81	0.50	0.07	9	66
	Petunidin 3-O-glucoside	Red grape and blueberry (juice)	-2.78	479.41	200.7	0.69	1.81	1.15	0.08	10	73
	Petunidin 3-O-glucoside	Red grape (juice)	-2.78	479.41	200.7	0.69	1.81	1.30	0.5	8	88
Flavanols	Epicatechin	Pure compound	1.37	290.27	110.37	0.85	1.08	1.00	0.50	9	59
	Epicatechin	Cranberry (juice)	1.37	290.27	110.37	0.85	1.08	2.60	0.76	10	76
	Epicatechin	Cocoa (drink)	1.37	290.27	110.37	0.85	1.08	2.00	NA	6	89
	Epicatechin	Green tea (drink)	1.37	290.27	110.37	0.85	1.08	0.78	0.19	12	82
	Epicatechin and catechin	Cocoa (drink)	1.37	290.27	110.37	0.85	1.08	1.50	NA	6	84
	Epicatechin gallate	Green tea (drink)	2.54	442.38	177.13	1.44	1.72	1.00	0.30	5	87
	Epigallocatechin	Green tea (drink)	1.08	306.27	130.6	0.84	1.01	0.72	0.12	12	82

	Epigallocatechin	Green tea (drink)	1.08	306.27	130.6	0.84	1.01	0.50	0.00	5	87
	Epigallocatechin gallate	Green tea (drink)	2.25	458.38	197.36	1.40	1.61	1.69	0.50	12	82
	Epigallocatechin gallate	Green tea (drink)	2.25	458.38	197.36	1.40	1.61	0.60	0.10	5	87
	Procyanidin B2 dimer	Cocoa (drink)	2.58	578.53	220.75	1.90	3.02	2.00	NA	6	89
Flavonols	Myricetin	Cranberry (juice)	1.39	318.24	151.58	0.91	0.91	1.70	0.41	10	76
	Quercetin	Cranberry (juice)	1.68	302.24	131.35	0.92	0.97	1.40	0.41	10	76
Hydroxy-benzoic acids	4-Hydroxybenzoic acid	Cranberry (juice)	1.37	138.12	57.53	0.63	0.60	0.80	0.22	10	76
	Protocatechuic acid	Montmorency tart cherry (juice)	0.88	154.12	77.75	0.60	0.56	1.00	NA	12	71
	Protocatechuic acid	Montmorency tart cherry (juice)	0.88	154.12	77.75	0.60	0.56	1.00	NA	12	71
	Vanillic acid	Cranberry (juice)	1.19	168.15	66.76	0.65	0.68	0.70	0.09	10	76
	Vanillic acid	Montmorency tart cherry (juice)	1.19	168.15	66.76	0.65	0.68	2.00	NA	12	71
	Vanillic acid	Montmorency tart cherry (juice)	1.19	168.15	66.76	0.65	0.68	1.00	NA	12	71
Hydroxy-cinnamic acids	Caffeic acid	Cranberry (juice)	0.94	180.16	77.75	0.64	0.67	0.80	0.16	10	76
	Ferulic acid	Cranberry (juice)	1.25	194.19	66.76	0.69	0.82	0.60	0.09	10	76
	p-Coumaric acid	Cranberry (juice)	1.43	164.16	57.53	0.67	0.72	0.60	0.09	10	76
	Sinapic acid	Cranberry (juice)	1.26	224.21	76	0.73	0.93	0.60	0.16	10	76

Hydroxy-phenylacetic acids	3,4-Dihydroxy-phenylacetic acid	Cranberry (juice)	0.39	168.15	77.75	0.57	0.62	0.80	0.22	10	76
Stilbenes	*cis*-Piceid (*cis*-Resveratrol 3-O-glucoside)	Red wine (drink)	1.2	390.39	139.84	1.01	1.67	1.13	0.15	10	83
	trans-Piceid (*trans*-Resveratrol 3-O-glucoside)	Red wine (drink)	1.2	390.39	139.84	1.01	1.67	0.86	0.19	10	83
Carotenoids	β-Carotene	Pure compound	9.843	536.89	0	11.12	16.43	7.00	0.83	8	72
	Fucoxanthinol	Kombu (extract)	8.49	658.92	96.36	9.44	16.22	4.00	NA	18	69
	Lutein	Marigold flower (extract)	9.307	568.89	40.46	10.05	14.28	19.00	2.05	8	72
	Lycopene	Tomato (drink)	9.977	536.89	0	11.59	16.43	5.00	NA	5	65
	Lycopene	Tomato (drink)	9.977	536.89	0	11.59	16.43	5.00	NA	5	65
	Lycopene	Tomato (drink)	9.977	536.89	0	11.59	16.43	5.00	NA	5	65
	Lycopene	Tomato (drink)	9.977	536.89	0	11.59	16.43	5.00	NA	5	65
Semi-solid intake											
Anthocyanins	Cyanidin 3-O-glucoside	Acai berry (pulp)	-2.79	449.39	191.46	1.15	1.11	2.17	0.11	12	77

	Cyanidin 3-O-glucoside	Red grape and blueberry (juice and puree)	-2.79	449.39	191.46	1.15	1.11	1.13	0.13	10	73
	Cyanidin 3-O-glucoside	Raspberry (homogenised whole fruit)	-2.79	449.39	191.46	1.15	1.11	1.00	NA	10	74
	Cyanidin 3-O-glucosylrutinoside	Montmorency tart cherry (whole fruit)	-4.79	757.67	329.54	2.91	3.91	2.00	NA	12	85
	Cyanidin 3-O-glucosylrutinoside	Montmorency tart cherry (whole fruit)	-4.79	757.67	329.54	2.91	3.91	4.00	NA	12	85
	Cyanidin 3-O-rutinoside	Montmorency tart cherry (whole fruit)	-3.49	595.53	250.39	1.52	2.17	1.50	NA	12	85
	Cyanidin 3-O-rutinoside	Montmorency tart cherry (whole fruit)	-3.49	595.53	250.39	1.52	2.17	2.00	NA	12	85
	Delphinidin 3-O-glucoside	Red grape and blueberry (juice and puree)	-3.08	465.39	211.69	1.29	1.03	1.12	0.13	10	73
	Malvidin 3-O-glucoside	Red grape and blueberry (juice and puree)	-2.47	493.44	189.7	1.03	1.69	1.25	0.13	10	73
	Peonidin 3-O-glucoside	Red grape and blueberry (juice and puree)	-2.49	463.42	180.47	1.03	1.43	1.15	0.10	10	73
	Petunidin 3-O-glucoside	Red grape and blueberry (juice and puree)	-2.49	463.42	180.47	1.15	1.32	1.18	0.17	10	73
Vitamins	Ascorbic acid	Kiwi fruit (whole fruit)	-1.4	176.12	107.22	0.75	0.24	2.00	NA	9	63
Solid intake											

Anthocyanins	Cyanidin 3-O-glucoside	Pure compound	-2.79	449.39	191.46	3.95	2.85	1.81	0.16	8	64
Flavanols	Epicatechin	Green tea (extract)	1.37	290.27	110.37	1.62	2.66	1.40	0.11	8	80
	Epicatechin	Green tea (extract)	1.37	290.27	110.37	1.62	2.66	1.65	NA	20	79
	Epicatechin gallate	Green tea (extract)	2.54	442.38	177.13	1.59	3.06	1.20	0.14	8	80
	Epicatechin gallate	Green tea (extract)	2.54	442.38	177.13	1.59	3.06	1.53	NA	20	79
	Epigallocatechin	Green tea (extract)	1.08	306.27	130.6	1.65	2.46	1.30	0.11	8	80
	Epigallocatechin	Green tea (extract)	1.08	306.27	130.6	1.65	2.46	1.64	NA	20	79
	Epigallocatechin gallate	Green tea (extract)	2.25	458.38	197.36	1.58	2.83	0.90	0.07	8	80
	Epigallocatechin gallate	Green tea (extract)	2.25	458.38	197.36	1.58	2.83	1.36	NA	20	79
Hydroxy-benzoic acids	Ellagic acid	Pomegranate (extract)	0.94	302.19	141.33	1.67	2.25	1.97	0.41	20	68
	Ellagic acid	Pomegranate (extract)	0.94	302.19	141.33	1.67	2.25	1.42	0.24	20	68
Stilbenes	Resveratrol	Pure compound	2.99	228.25	60.68	1.63	2.94	1.58	0.27	15	58
	trans-Resveratrol	Pure compound	2.99	228.25	60.68	1.63	2.94	1.12	0.26	12	81
	cis-Piceid (cis-Resveratrol 3-O-glucoside)	Pure compound	1.2	390.39	139.84	1.63	3.23	1.00	0.06	10	83
	trans-Piceid (trans-Resveratrol 3-O-glucoside)	Pure compound	1.2	390.39	139.84	1.63	3.23	0.88	0.08	10	83
Carotenoids	β-Carotene	Pure compound	9.843	536.89	0	15.27	15.45	36.00	NA	12	91

	β-Carotene	Pure compound	9.843	536.89	0	15.27	15.45	36.00	NA	12	91
	β-Carotene	Pure compound	9.843	536.89	0	15.27	15.45	35.00	NA	12	91
	Crocetin	Pure compound	4.63	328.41	74.6	2.01	3.98	4.8	0.32	10	90
	Crocetin	Pure compound	4.63	328.41	74.6	2.01	3.98	4	0.28	10	90
	Crocetin	Pure compound	4.63	328.41	74.6	2.01	3.98	4.6	0.32	10	90
	Lutein	Pure compound	9.307	568.89	40.46	11.27	13.23	36.8	4.15	18	61
Vitamins	Ascorbic acid	Pure compound	-1.4	176.12	107.22	2.53	1.72	3.00	NA	9	63
	Calcitriol	Pure compound	5.56	416.65	60.68	2.48	6.25	4.00	NA	10	60
	Phenprocoumon	Pure compound	4.09	280.32	50.44	1.83	3.89	2.25	NA	24	70
	Phylloquinone	Pure compound	8.803	450.71	34.14	8.64	8.62	8.8	0.38	20	86

* calculated using the Molinspiration Chemoinformatics calculator

** calculated from the predictive model

collected from the literature

S3 Table. The PHv_fasted dataset for validation of the predictive model. All compounds were taken as solid form.

Pharma-ceutical compound	LogP*	M_r*	PSA* ($Å^2$)	Predicted T_{max}_LogP** (h)	Predicted T_{max}_PSA** (h)	Measured T_{max}#(h)			Reference#
						Mean	SE	N	
Nadolol	1.15	309.41	81.95	1.64	3.50	3.00	1	10	123
Nifedipine	3.07	346.34	110.46	1.64	3.33	2.80	0.5	20	101
Odanacatib	3.96	525.57	99.06	1.80	7.38	3.00	1	44	136
Tramadol	3.18	263.38	32.70	1.65	4.11	2.20	0.8	20	96
Amisulpride	1.56	369.49	101.74	1.60	3.88	2.30	1.9	8	111
Levofloxacin	-0.26	361.37	75.01	1.96	4.53	1.84	0	12	139
Mirodenafil	3.23	531.68	120.77	1.66	6.50	3.25	2.75	6	124
Talinolol	3.36	363.50	86.11	1.68	4.23	2.10	0.81	14	142
Aleglitazar	4.73	437.52	81.80	2.05	5.86	3.00	0	6	137
Mitiglinide	3.37	315.41	57.61	1.68	4.26	0.36	0.16	8	144
Risperidone	2.96	410.49	64.17	1.62	5.95	1.20	0.7	25	125
Sonidegib	5.89	485.51	63.70	2.72	8.05	3.00	1	6	147
Imidafenacin	2.34	319.01	60.92	1.58	4.22	1.50	0.45	6	127
Olanzapine	3.47	312.44	35.16	1.70	4.92	4.92	1.8	6	112
Imatinib	3.89	493.62	86.28	1.78	7.10	3.63	1.2	30	129
Arbidol	4.86	477.42	54.71	2.10	8.30	0.75	0.75	20	119
Ruxolitinib	1.83	306.37	83.19	1.59	3.43	1.00	0.5	16	134
Risperidone	2.96	410.49	64.17	1.62	5.95	1.00	0.31	10	120
Ritonavir	6.93	720.96	145.78	3.81	11.63	4.20	2.2	10	135
Rosuvastatin	2.11	481.55	140.92	1.58	4.62	2.46	0.65	12	117
Capravirine	4.45	451.38	83.05	1.95	6.14	3.00	1.5	5	99
Proguanil	2.05	253.74	83.79	1.58	2.77	2.80	1.2	15	135
CH 4987655	3.22	565.29	100.12	1.66	8.59	1.00	1	40	115
RO 5068760	4.11	647.44	128.20	1.84	9.80	2.00	2	6	114
Tinidazole	-0.06	247.28	97.79	1.89	2.45	2.15	0.47	50	100
Pantoprazole	1.95	383.38	86.35	1.58	4.57	2.27	0.52	28	103
Moxifloxacin	0.39	401.44	83.80	1.77	5.00	1.00	1.5	16	116
Tacrolimus	4.26	804.03	178.38	1.88	12.90	1.37	0.64	15	97
Ethionamide	1.46	166.25	38.92	1.61	2.67	1.70	0.87	16	92

Metopimazine	2.73	445.61	85.41	1.60	5.90	0.87	0.5	6	110
Sulfonamide	-0.29	172.21	86.19	1.97	1.97	1.98	0.51	8	122
Ketoconazole	3.77	531.44	69.08	1.75	9.32	1.40	0.1	10	121
Miconazole	5.72	416.14	27.06	2.59	7.89	2.60	0.3	12	121
Nifedipine	3.07	346.34	110.46	1.64	3.33	1.00	0	10	126
Spironolactone	3.03	416.58	3.03	1.63	9.35	1.70	1	9	128
Ciramadol	2.56	249.35	43.69	1.59	3.60	0.90	1	14	93
Cyclosporin	3.61	1202.64	278.78	1.72	31.47	2.00	2	11	118
S 3304	2.80	464.57	99.26	1.61	5.77	2.00	1	6	140
MK 462	1.39	269.35	49.75	1.61	3.74	1.60	1.2	12	102
Itraconazole	5.32	705.65	104.73	2.34	14.58	3.90	1	28	95
Indinavir	2.51	613.80	118.02	1.59	9.20	0.90	0.5	10	143
Celecoxib	3.61	381.38	77.99	1.72	4.80	2.44	0.83	24	130
Rifampin	2.62	822.95	220.16	1.60	10.39	2.18	1.44	14	132
Topiramate	0.16	339.37	115.57	1.83	3.12	1.75	1.17	28	105
Ethambutol	0.35	204.31	64.51	1.78	2.60	2.46	0.86	14	133
Isoniazid	-0.97	137.14	68.01	2.27	1.94	1.02	1.1	14	148
Cilostazol	3.40	369.47	3.40	1.68	7.72	3.00	3	23	98
Febuxostat	3.68	316.38	83.22	1.74	3.57	0.80	0.6	24	113
Apixaban	1.78	459.51	110.77	1.59	5.22	3.00	3	43	108
Ziprasidone	4.05	412.95	48.47	1.82	6.70	3.60	0.7	8	109
Cefprozil	-1.68	389.43	132.96	2.74	3.38	1.20	0.8	12	94
Eprosartan	4.89	424.52	92.42	2.12	5.16	1.50	1.5	6	138
Amoxicillin	-1.35	365.41	132.86	2.50	3.07	1.86	0.3	16	106
Ampicillin	-0.87	349.41	112.73	2.22	3.32	1.49	0.5	16	106
Pyrazinamide	-0.71	123.12	68.88	2.14	1.82	1.71	1.19	14	131
Bosentan	4.16	551.63	145.67	1.85	5.91	3.50	4.5	16	104
Telbivudine	-1.43	242.23	104.56	2.55	2.29	3.00	3	24	145
Tenoxicam	0.76	337.38	99.60	1.70	3.46	1.70	1.2	6	107
Eltrombopag	5.12	442.48	116.82	2.23	4.67	3.50	2	25	151
Fluconazole	-0.12	306.28	81.66	1.91	3.47	3.08	0.79	12	146

* calculated using the Molinspiration Chemoinformatics calculator

** calculated from the predictive model

collected from the literature

S4 Table. The Pharmaceutical fed dataset for validation of the predictive model. All compounds were taken as solid form.

Pharma-ceutical compound	LogP*	M_r*	PSA* ($Å^2$)	Predicted T_{max}_LogP** (h)	Predicted T_{max}_PSA** (h)	Measured T_{max}#(h)			Reference#
						Mean	SE	N	
Odanacatib	3.96	525.57	99.06	1.80	7.38	4	1	44	136
Amisulpride	1.56	369.49	101.74	1.60	3.88	1.7	0.6	8	111
Mitiglinide	3.37	315.41	57.61	1.68	4.26	1.97	0.81	8	144
Rosuvastatin	2.11	481.55	140.92	1.58	4.62	4.28	1.35	8	117
Pantoprazole	1.95	383.38	86.35	1.58	4.57	6.287	4.41	28	103
Moxifloxacin	0.39	401.44	83.80	1.77	5.00	2.5	1.5	16	116
Tacrolimus	4.26	804.03	178.38	1.88	12.90	6.47	3.04	15	97
Ethionamide	1.46	166.25	38.92	1.61	2.67	2.6	0.94	16	92
Metopimazine	2.73	445.61	85.41	1.60	5.90	1.5	0.5	6	110
Sulfonamide	-0.29	172.21	86.19	1.97	1.97	2.22	1.07	8	122
Ketoconazole	3.77	531.44	69.08	1.75	9.32	2.3	0.3	12	121
Nifedipine	3.07	346.34	110.46	1.64	3.33	3.5	0.5	10	126
Spironolactone	3.03	416.58	3.03	1.63	9.35	1.4	1.1	9	128
Ciramadol	2.56	249.35	43.69	1.59	3.60	2.1	2.1	14	93
S 3304	2.80	464.57	99.26	1.61	5.77	5	1	6	140
MK 462	1.39	269.35	49.75	1.61	3.74	2.9	1.4	12	102
Itraconazole	5.32	705.65	104.73	2.34	14.58	4.5	1.1	28	95
Indinavir	2.51	613.80	118.02	1.59	9.20	2.8	1.9	16	143
Celecoxib	3.61	381.38	77.99	1.72	4.80	3.42	1.28	24	130
Rifampin	2.62	822.95	220.16	1.60	10.39	4.43	1.12	14	132
Ethambutol	0.35	204.31	64.51	1.78	2.60	3.21	1.34	14	133
Isoniazid	-0.97	137.14	68.01	2.27	1.94	1.93	1.62	14	148
Cilostazol	3.40	369.47	3.40	1.68	7.72	3.5	1.5	23	98
Febuxostat	3.68	316.38	83.22	1.74	3.57	1.9	0.9	24	113
Apixaban	1.78	459.51	110.77	1.59	5.22	4	5	43	108
Ziprasidone	4.05	412.95	48.47	1.82	6.70	4.5	1.4	8	109
Cefprozil	-1.68	389.43	132.96	2.74	3.38	2	1.5	12	94
Eprosartan	4.89	424.52	92.42	2.12	5.16	3	1.5	4	138
Artemisinin	3.32	282.34	54.01	1.67	3.82	1.78	1.23	16	150

Amoxicillin	-1.35	365.41	132.86	2.50	3.07	2.4	0.41	16	106
Ampicillin	-0.87	349.41	112.73	2.22	3.32	2.48	0.74	14	106
Pyrazinamide	-0.71	123.12	68.88	2.14	1.82	3.09	1.75	16	131
Bosentan	4.16	551.63	145.67	1.85	5.91	4	4	24	104
Telbivudine	-1.43	242.23	104.56	2.55	2.29	3	1	24	145
Posaconazole	4.33	700.79	115.72	1.91	13.24	5.5	1.76	20	149
Tenoxicam	0.76	337.38	99.60	1.70	3.46	3.8	2	6	107
Eltrombopag	5.12	442.48	116.82	2.23	4.67	4	8	25	151
Fluconazole	-0.12	306.28	81.66	1.91	3.47	3.5	1	12	146

[*] calculated using the Molinspiration Chemoinformatics calculator

[**] calculated from the predictive model

[#] collected from the literature
